# Risk communication about high‐dose MDMA: Impact of a hypothetical drug alert on future MDMA use

**DOI:** 10.1111/dar.14037

**Published:** 2025-03-07

**Authors:** Joel Keygan, Breanna Willoughby, Raimondo Bruno, Monica J. Barratt, Amy Peacock

**Affiliations:** ^1^ National Drug and Alcohol Research Centre UNSW Sydney Sydney Australia; ^2^ School of Psychology and Public Health, Centre of Alcohol Policy Research La Trobe University Melbourne Australia; ^3^ School of Health and Biomedical Sciences RMIT University Melbourne Australia; ^4^ School of Psychological Sciences University of Tasmania Hobart Australia; ^5^ Criminology and Justice Studies, Social Equity Research Centre and Digital Ethnography Research Centre RMIT University Melbourne Australia

**Keywords:** behaviour change, drug alerts, harm reduction, MDMA, risk communication

## Abstract

**Introduction:**

Despite high‐dose 3,4‐methylenedioxymethamphetamine (MDMA) drug alerts being distributed, no research has been conducted as to changes in use in response. This study aimed to determine if: (i) high‐dose MDMA drug alerts, and (ii) varied descriptions of dose, effects and actions to reduce harm were associated with intentions to reduce the initial MDMA dose in a hypothetical scenario.

**Methods:**

Australians who used MDMA pills/capsules in the past year completed an online survey. Respondents were randomised into alert (*n* = 441) or control (*n* = 184) conditions, with the former receiving a high‐dose MDMA alert with systematically varied descriptions of dose, effects and actions to reduce harm. Multinomial logistic regressions determined the association between receipt of drug alert (and varying alert content) and hypothetical MDMA dosing.

**Results:**

Almost half (45.4%) of those in any alert condition reported intention to not use (20.7% of control participants) and 46.7% stated they would use and reduce their initial dose (69.0% of control group). Compared to the control group, those who received an alert were significantly more likely to report intention to not use the drug, as compared to taking a smaller initial dose (adjusted relative risk ratio [aRRR] = 3.28, 95% confidence interval [CI] 2.13, 5.07) or taking the same/higher initial dose (aRRR = 2.62, 95% CI 1.31, 5.22). There was no significant association between different alert phrasing and intended behaviour.

**Discussion and Conclusions:**

While there was no significant effect of variation in phrasing, receipt of an alert promoted intended harm reduction behaviours. Future research assessing actual behaviour and different substances (e.g., heroin, methamphetamine) is important to further understand the utility of this public health communication.

**Key Points**

1. People exposed to a drug alert about high‐dose MDMA were more likely to report an intention to avoid using the drug compared with those informed about potentially possessing high‐dose MDMA without an alert.
2. Altering information on dose, effects and harm‐reduction strategies did not significantly change responses.
3. Drug alerts about higher‐risk substances may be an effective tool for government and other health agencies to encourage harm reduction behaviour among people who use drugs, although a study of actual behavioural outcomes in response to alerts about different substances is warranted.

## 1 INTRODUCTION

3,4‐methylenedioxymethamphetamine (MDMA) is a stimulant drug with entactogenic properties that produces euphoric, prosocial and energising effects [1]. MDMA is the primary psychoactive chemical in ‘ecstasy’ most commonly available in the form of pills, capsules, powder and crystal [2], all of which can contain variable amounts of MDMA and can be adulterated with or substituted for other compounds [3, 4, 5]. Almost all (94%) of people who used MDMA reported oral administration, while 26% reported nasal administration (snorting), as found in a recent large Australian study [2]. While most people who use MDMA do not experience serious adverse effects, MDMA toxicity can result in significant clinical presentations (e.g., hyperthermia, serotonin syndrome, stroke and hyponatremia) and, in some cases, death [6, 7, 8]. It should be noted that MDMA now has recognised medical uses in the context of psychotherapy to treat post‐traumatic stress disorder [9] and it is now possible to administer in a controlled environment with a known dose under psychiatric supervision in Australia [10].

Rigg and Sharp [8] reviewed factors that contribute to MDMA‐related harms, including declining price, unpredictable quality, media attention, hyperthermia, hyponatremia and drug interactions. Environmental factors related to the use of MDMA at dance festivals and nightclubs are also critical: including crowding, hot temperature, dehydration and exhaustion (particularly at multi‐day events) [11]. Additionally, individual factors affect how MDMA is metabolised: these include CYP2D6 activity, weight, sex and tolerance [12]. Considered alongside and moderated by these contextual and individual factors, MDMA dose is an important modifiable risk factor. For example, in a Dutch study of self‐reported effects linked with analysed MDMA pills, doses of >120 mg were associated with adverse effects [3]. A common dose when orally consumed is 80–120 mg, according to crowd‐sourced Psychonaut Wiki [13].

Since 2010 (following a period of reduced MDMA availability and purity), MDMA availability has increased, new forms (e.g., powder and crystal) have become more common, and the MDMA content of tablets has become higher and more variable [14, 15, 16, 17]. In particular, the circulation of high‐dose MDMA tablets, defined here as tablets containing 200 mg or more (nearly twice the median dose in Australia; [17]), has caused concern because of the risk of adverse effects, with tablets containing 270–340 mg being identified in some countries [15, 18]. Indeed, increases in MDMA‐related deaths in Australia [19, 20, 21] and internationally [6, 8] have been observed over a similar period.

Harm reduction behaviours such as taking smaller doses, not mixing substances and spacing out doses can reduce the risk of negative effects when taking MDMA [22, 23, 24]. Employment of many of these strategies relies on information on the substance contents and dose, and research shows that people who use drugs want more information about the contents of their drugs [25, 26]. Indeed, Measham et al. [27, 28, 29] found that people who received results from a drug checking service that showed their drug contained a higher MDMA content than anticipated reported an intention to take a smaller dose. However, the lack of access to government‐sanctioned drug checking services, and particularly fixed‐site services,^1^ across most of Australia means that people mainly obtain objective information on dose from: (i) colorimetric reagent kits, which are limited in their capacity to quantify dose [30]; or (ii) public health agencies engaging in risk communication, disseminating information on health risks from particular substances [31, 32].

Drug alerts are a form of risk communication, whereby public notices from health agencies are released notifying of adulterated substances or changes in potency [33, 34, 35, 36]. To date, research assessing the influence of high‐dose drug alerts on harm reduction behaviours has typically focused on people who inject drugs. For example, alerts about high‐strength heroin had limited effectiveness in British Columbia, with information presented overshadowed by the desire to obtain the ‘advertised’ high‐quality heroin [37]. The little research conducted to date suggests the use of brief messages with clear, simple language, communicated in a timely manner and disseminated through media may increase alert effectiveness [31, 33, 34, 35, 36, 38].

Within Australia, public health agencies in various jurisdictions (e.g., New South Wales, Victoria, Australian Capital Territory, South Australia and Queensland) have released high‐dose MDMA drug alerts. In the year 2024, for example, 15 high‐dose MDMA alerts were issued across Australia according to The Know, a site that collates alerts nationally, and many of these individual alerts included information about multiple high‐dose MDMA tablets [39]. Currently, there is no research (locally or internationally, to the best of our knowledge) systematically assessing behaviour in response to alerts about high‐dose MDMA among people who use drugs. Given reports of circulating high‐dose MDMA continuing in Australia and elsewhere, it is critical to conduct research to understand the potential impact of these alerts on future behaviour. As such, this study aimed to determine if: (1) a hypothetical MDMA high‐dose drug alert; and (2) varied descriptions of (i) dose, (ii) effects and (iii) actions to reduce harm within the drug alert were associated with intentions to reduce initial MDMA dose.

## 2 METHODS

### 2.1 Study design, participants and procedure

Overall, this study was a simple intervention (drug alert) versus control comparison. The drug alerts, however, had 18 different manipulations, using systematic factorial combinations of three levels of description of MDMA dose, two levels of description of MDMA effects and three levels of suggestions of actions to reduce harm. Participants were randomised into an oversampled control group or one of the 18 intervention groups. The control group was oversampled because we planned to compare the combined intervention group (any of the 18 levels of alert) with the control group (Aim 1).

Participants (>18 years and older) who lived in Australia, had access to the internet, and had used ‘MDMA/ecstasy’ in the previous 12 months were recruited between June and early August 2020 from Facebook advertisements. After providing consent, participants completed an online survey administered via Qualtrics that took approximately 30 min to complete. Intervention group participants were exposed to 1 of 18 drug alert variants, with control participants receiving no alert. The 18 separate drug alerts were put into a simple randomisation sequence on Qualtrics with a 1:1 allocation ratio (e.g., each alert was allocated one participant, and then the cycle began again, so that no alert ever had more than one person extra allocated relative to another alert). On completion, participants could enter the prize draw to win an Apple iPad. Ethical approval was granted by the University of New South Wales Human Research Ethics Committee (HC200294).

### 2.2 Materials and measures

#### 2.2.1 Independent variables

**FIGURE 1:**
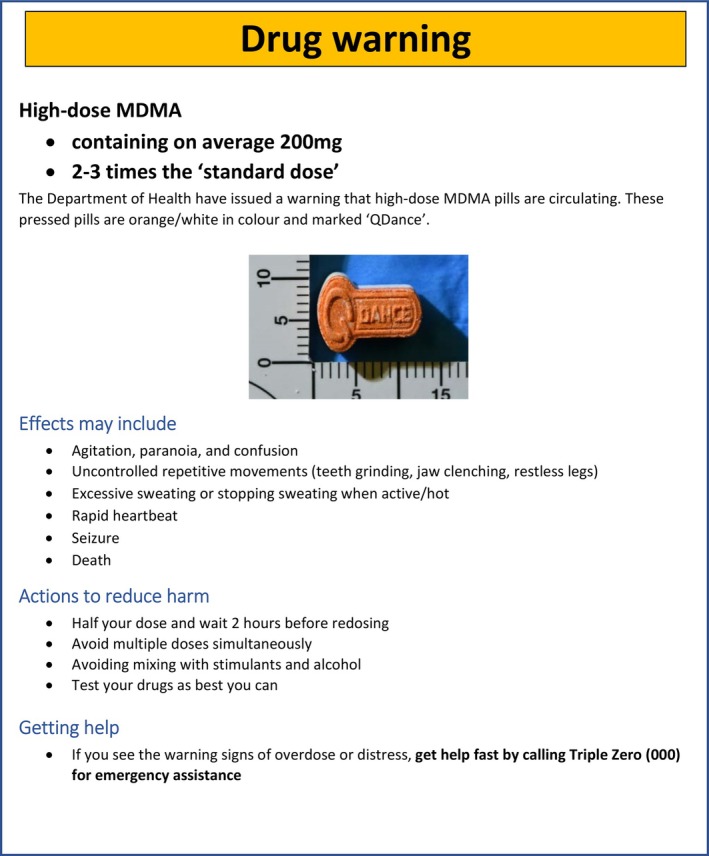
An example of one version of the drug alert shown to participants. The following text was included prior to the image: “Now I want you to imagine that you have just seen the below post on a Facebook page of The Loop, an organisation that represents people who use drugs in Australia”.

Intervention participants were shown 1 of 18 high‐dose MDMA drug alerts, reflecting each combination of the three independent variables: (i) dose (three levels); (ii) effects (two levels); and (iii) actions to reduce harm (three levels) (see Figure 1 for an example of the drug alert). The three descriptions of ‘dose’ varied in quantification of high‐dose MDMA; specifically: (i) ‘high‐dose MDMA’; (ii) ‘high‐dose MDMA containing on average 200mg’; or (iii) ‘high‐dose MDMA containing on average 200mg, which is 2–3 times the “standard dose”’. The two descriptions of ‘effects’ varied as to information on potential seizure/fatality: (i) ‘effects may include: agitation, paranoia and confusion; uncontrolled repetitive movements (e.g., teeth grinding, jaw clenching, restless legs); excessive sweating or stopping sweating when active/hot; and rapid heartbeat’ or (ii) ‘effects may include: agitation, paranoia and confusion; uncontrolled repetitive movements (e.g., teeth grinding, jaw clenching, restless legs); excessive sweating or stopping sweating when active/hot; rapid heartbeat; seizure; and death’. The three descriptions of ‘actions to reduce harm’ varied as to information on abstinence versus harm reduction strategies, specifically: (i) emergency service advice as follows ‘If you see the warning signs of overdose or distress, get help fast by calling Triple Zero (000) for emergency assistance’; (ii) emergency service advice and ‘Do not take this drug at all’; or (iii) emergency service advice and the following: ‘half your dose and wait 2 hours before redosing; avoid multiple doses simultaneously; avoiding mixing with stimulants and alcohol; test your drugs as best you can’. This content about dose, effects and actions to reduce harm was informed by studying information within existing alerts issued in Australia [39]. All other elements of the alert were held consistent across intervention groups, including contextual information presented before the alert stating that the following was a ‘post on the Facebook page of The Loop, an organisation who represents people who use drugs in Australia’.^2^ Control participants were instead shown a message thanking them for answering questions regarding their MDMA history and then proceeded to the subsequent stage of the survey.

#### 2.2.2 Outcome variable

All participants answered questions pertaining to acquiring and ingesting high‐dose MDMA and harm reduction strategies. Intervention participants were asked to imagine a hypothetical scenario where they had MDMA/ecstasy pills/capsules that they thought might match those described in the alert. Control participants were instead asked to imagine a hypothetical situation where they possessed MDMA/ecstasy pills/capsules that may contain twice the standard dose of MDMA. Participants were first asked if they would consume the drug. Response options for intervention participants were: ‘yes’, ‘no, because I saw the alert’, ‘no, because I prefer to use MDMA crystal/powder’, ‘no, because of other reasons’, and ‘I don't know’. Response options for control participants were: ‘yes’, ‘no’, ‘no, because I prefer to use MDMA crystal/powder’, and ‘I don't know’.

Intervention and control participants who said they would use the drug were then asked what initial dose of pills/capsules they would consume, with instruction that the drug may be two times stronger than normal, and a prompt reporting their earlier response as to historical initial dose in a typical session in the past 12 months (asked prior to the alert for the intervention groups). Response options for both historical initial dose and future intended initial dose were categorical, increasing in quarter amounts until one tablet, and then in half amounts until ‘>6 pills/capsules’. The outcome variable was computed in two steps. First, intended initial dose (asked after alert exposure or control scenario) was subtracted from self‐reported typical initial dose in the past 12 months (asked prior to alert exposure or control scenario) for those who reported intention to use; a positive value was considered to be an intention to reduce their initial dose. A categorical variable was then computed for use in analysis classifying participants by their intention to: (i) not use because of the alert; (ii) use and reduce initial dose; (iii) use and not reduce initial dose of high‐dose MDMA (i.e., intended dose was equivalent or greater than typical dose); (iv) not use for another reason/because they prefer powder/crystal; and (v) do not know.

#### 2.2.3 Covariates

Covariates comprised gender, age, lifetime frequency of MDMA use, lifetime experience of any MDMA non‐fatal overdose and awareness of any public notices (drug alerts) about high‐dose MDMA in the last 12 months. Covariates were selected based on the literature on MDMA/ecstasy use and harms and on drug alerts (see Table S1, Supporting Information, for full description of covariates and rationale for their inclusion) and were assessed prior to independent and outcome variables.

### 2.3 Data analysis

Participant data were imported into a range of statistical programs, including SPSS (Version 26), Jamovi (Version 1.6.3) and Stata/SE (Version 16.1), depending on the required analysis.

Before analyses, data were cleaned to remove cases who were ineligible or did not respond to core survey items (see Figure S1). There were 2146 participants who started the survey, of whom 1664 were eligible and proceeded; of these, 1039 were excluded from the final sample, the majority because they exited the survey before completing the outcome variables (*n* = 685), and others because their responses (e.g., ‘I don't know’ (*n* = 80) or ‘prefer crystal/powder’ (*n* = 241) to the outcome variable) were not applicable to answering the research question (final sample *n* = 625).

Descriptive statistics comprised mean and standard deviation for continuous variables, median and interquartile range for count or skewed variables, and percentages for categorical variables. Chi‐square analyses were conducted to determine whether covariates were associated with the outcome variable (Table S2). For Aim 1, hierarchical multinomial logistic regressions were conducted to identify associations between alert exposure (or no exposure) and the outcome, reported as relative risk ratios with 95% confidence intervals. The alert exposure variable only was included at step 1; this variable plus those covariates significant at *p* < 0.25 in chi‐square analyses were included at step 2. The stepwise regression excluded variables that were *p* > 0.25, as Hosmer and Lemeshow [40] demonstrated that using *p* < 0.05 in this step can result in an overly conservative model. Age, gender and their interaction term were included regardless of statistical significance, as there is existing research demonstrating the known importance of these factors for this study (see Table S1). The model was run twice to enable comparison across the three levels of the final outcome variable: once with ‘use and reduce initial dose’ as the referent category, and once with ‘use and do not reduce initial dose’ as the referent category. For Aim 2, a three‐step hierarchical binary logistic model was created to determine: (i) the association between each of the three drug alert content independent variables on the outcome in isolation; (ii) the association between each drug alert content independent variable on the outcome controlling for covariates significant at *p* < 0.25 in chi‐square analyses; and (iii) the association between each drug alert content independent variable on the outcome controlling for covariates and the other two drug alert content independent variables, reported as odds ratios with 95% confidence intervals. Associations are interpreted where *p* < 0.05. Complete case analysis was used; number of missing responses per covariate is reported.

## 3 RESULTS

Of the 625 participants, the majority were male (58.6%), with a median age of 22 (interquartile range 19–25; range 18–72). Most had used MDMA more than 10 times in their lifetime (67.5%). Three in five had never experienced an MDMA overdose (59.4%). The majority resided in Victoria (27.5%), New South Wales (26.1%) or Queensland (21.8%), and one‐third (31.4%) reported seeing an alert about high‐dose MDMA in the past 12 months. Two in three (65.2%) endorsed 200 mg as a high dose of MDMA; 16.2% did not (see Table 1 for full sample characteristics).

**TABLE 1:** Demographic and MDMA use characteristics.

Variable	Total sample (*n* = 625), % (*n*)
Gender^a^	*n* = 625
Male	58.6 (366)
Female	41.4 (259)
Age, years	*n* = 625 Median 22 (IQR 19–25)
State/territory of residence	*n* = 625
Australian Capital Territory	1.9 (12)
New South Wales	26.1 (163)
Queensland	21.8 (136)
Northern Territory	0.2 (1)
South Australia	6.9 (43)
Tasmania	4.0 (25)
Victoria	27.5 (172)
Western Australia	11.7 (73)
Highest level of school	*n* = 616
Year 11 and below	11.2 (69)
Year 12 or equivalent	88.8 (547)
Employment status	*n* = 622
Employed	46.6 (290)
University student	34.6 (215)
Unemployed	8.8 (35)
Trade apprentice	3.1 (19)
TAFE student	2.4 (15)
Home duties	1.9 (12)
Other	2.6 (16)
Number of occasions of MDMA use (lifetime)	*n* = 625
1–10	32.5 (203)
11–50	42.1 (263)
≥51	25.4 (159)
Lifetime MDMA overdose	*n* = 625
Yes	33.9 (212)
No	59.4 (371)
I don't know	6.7 (42)
Awareness of drug alerts about high‐dose MDMA in last 12 months	*n* = 625
Yes	31.4 (196)
No	63.8 (399)
I don't know	4.8 (30)
Perceive 200 mg to be a high dose of MDMA	*n* = 625
Yes	65.2 (408)
No	16.2 (101)
I don't know	18.6 (116)

Abbreviations: IQR, interquartile range; MDMA, 3,4‐methylenedioxymethamphetamine.

^a^ Non‐binary/other gender was mentioned but excluded from the final sample due to low numbers.

### 3.1 Was there an effect of receiving alerts on intended MDMA use and initial dose? (Aim 1)

**FIGURE 2:**
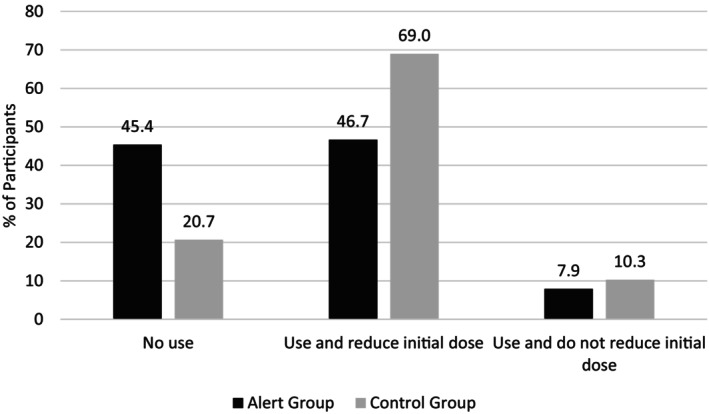
Reported 3,4‐methylenedioxymethamphetamine (MDMA) use and initial dose intentions for alert and control conditions.

Figure 2 shows the percentage of participants in the alert and control groups by reported intended behaviour. At step 1 of the hierarchical logistic regressions, the alert/control variable was entered separately (Table 2). Respondents exposed to any of the alerts were over three times more likely than the control group to report that they would avoid the drug altogether compared to using and reducing the initial dose. Similarly, they were over two times more likely than the control group to report that they would avoid the drug altogether compared with taking the same or a higher initial dose. The alert group was no more likely than the control group to report that they would take the same or a higher initial dose compared to using and reducing the initial dose.

**TABLE 2:** Hierarchical multinomial logistic regression models of association between alert receipt and intended MDMA use/initial dose (*n* = 625).

	‘No use’ versus ‘use and reduce initial dose’ (ref)^a^		‘Use and do not reduce initial dose’ versus ‘use and reduce initial dose’ (ref)^a^		‘No use’ versus ‘use and do not reduce initial dose’ (ref)^b^	
Variable	RRR (95% CI)	*p*	RRR (95% CI)	*p*	RRR (95% CI)	*p*
Step 1						
Condition (Alert)	**3.24 (2.15–4.90)**	**<0.001**	1.14 (0.62–2.07)	0.678	**2.86 (1.48–5.51)**	**0.002**
Step 2						
Condition (Alert)	**3.28 (2.13–5.07)**	**<0.001**	1.26 (0.68–2.33)	0.472	**2.62 (1.31–5.22)**	**0.006**
Gender (male)	**0.11 (0.02–0.55)**	**0.007**	0.01 (0.00–1.54)	0.075	8.49 (0.07–1047)	0.384
Age (continuous)^c^	0.98 (0.92–1.03)	0.384	**0.76 (0.62–0.94)**	**0.012**	**1.28 (1.04–1.59)**	**0.022**
Gender × age	1.06 (0.98–1.13)	0.122	1.24 (0.99–1.56)	0.062	0.85 (0.67–1.06)	0.162
Lifetime MDMA use						
1–10 times	**2.37 (1.56–3.58)**	**<0.001**	1.13 (0.51–2.47)	0.763	2.10 (0.94–4.67)	0.070
11–50 times			−	−		
51+ times	0.72 (0.44–1.17)	0.181	**2.39 (1.22–4.71)**	**0.012**	**0.30 (0.14–0.64)**	**0.002**

*Note*: *N* = 625. ‘Use and do not reduce initial dose’ *n* = 54, ‘Use and reduce initial dose’ *n* = 333, ‘No use’ *n* = 238. *p* < 0.05 are bolded.

Abbreviations: CI, confidence interval; MDMA, 3,4‐methylenedioxymethamphetamine; RRR, relative risk ratio; −, referent category.

^a^ The model was run twice: ‘Use and reduce initial dose’ as the referent category.

^b^ The model was run twice: ‘Use and do not reduce initial dose’ as the referent category.

^c^ Age squared was included but was removed as *p* > 0.25.

At step 2, covariates identified as significant through chi‐square analysis (see Table S2) were added into the model. The adjusted relative risk ratios for the association between alert/control group and intended use did not meaningfully change once these covariates were added.

### 3.2 Were there effects of varying alert content on decisions regarding intended MDMA use and initial dose? (Aim 2)

**FIGURE 3:**
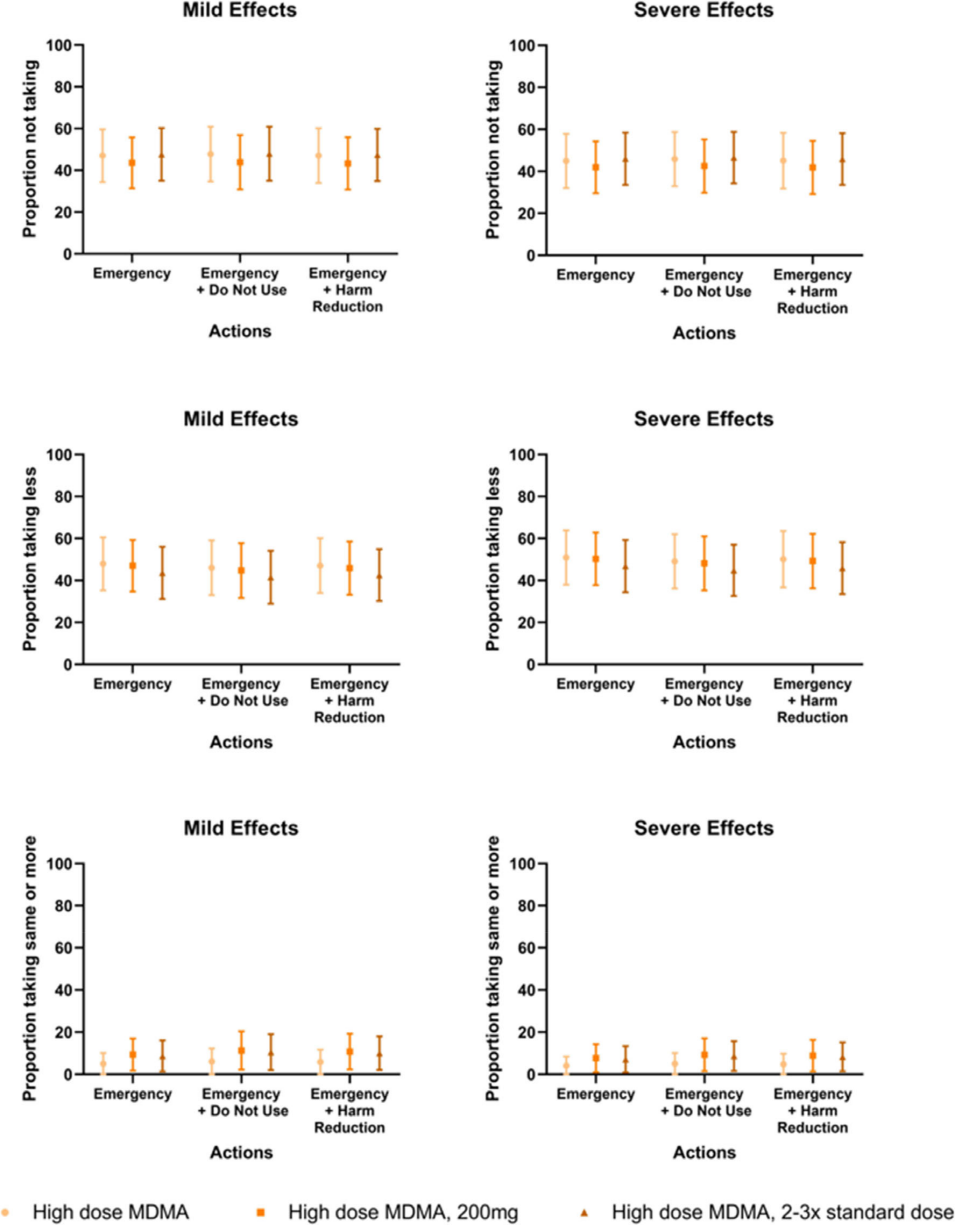
Reported 3,4‐methylenedioxymethamphetamine (MDMA) use and dose intentions for variations in drug alert conditions.

Figure 3 shows the unadjusted percentages reporting the behavioural outcome for each of the drug alert independent variables. Those responding ‘use and do not reduce initial dose’ were removed from regression analyses due to small cell size. Hierarchical binary logistic regression showed no significant associations between the three aspects of alert content (dose, effects and actions to reduce harm) and MDMA dosing behaviour (no use vs. use and reduce initial dose) at any step of the models (Table 3; see Table S3, for full model results).

**TABLE 3:** Hierarchical binary logistic regression models of association between alert content and intended behaviour (*n* = 406).

	Total sample (*n* = 406) % (*n*)	No use (ref) 49.3% (*n* = 200) % (*n*)	Use and reduce initial dose 50.7% (*n* = 206) % (*n*)	Step 1^a^ OR (95% CI, *p*)	Step 2^b^ AOR (95% CI, *p*)	Step 3^c^ AOR (95% CI, *p*)
Description of dose						
‘High dose MDMA’	31.8 (129)	31.5 (63)	32.0 (66)	–	–	–
‘High dose MDMA 200 mg’	32.8 (133)	31.5 (63)	34.0 (70)	1.06 (0.65–1.72, 0.812)	1.24 (0.74–2.01, 0.414)	1.24 (0.73–2.09, 0.425)
’High dose MDMA 200 mg 2–3× standard dose’	35.5 (144)	37.0 (74)	34.0 (70)	0.90 (0.56–1.45, 0.674)	1.11 (0.66–1.86, 0.688)	1.11 (0.66–1.86, 0.703)
Description of effects						
Mild	49.3 (200)	50.5 (101)	48.1 (99)	–	–	–
Severe	50.7 (206)	49.5 (99)	51.9 (107)	1.10 (0.75–1.63, 0.623)	1.07 (0.70–1.63, 0.766)	1.06 (0.70–1.62, 0.778)
Description of actions						
Emergency	35.5 (144)	35.0 (70)	35.9 (74)	–	–	–
Emergency + do not use	32.0 (130)	32.5 (65)	31.6 (65)	0.95 (0.59–1.52, 0.818)	0.94 (0.57–1.57, 0.817)	0.94 (0.57–1.57, 0.818)
Emergency + harm reduction	32.5 (132)	32.5 (65)	32.5 (67)	0.98 (0.61–1.56, 0.917)	1.03 (0.62–1.72, 0.904)	1.03 (0.62–1.71, 0.919)

*Note*: *p* < 0.05 are bolded.

Abbreviations: AOR, adjusted odds ratio; CI, confidence interval; MDMA, 3,4‐methylenedioxymethamphetamine; OR, odds ratio; − referent category.

^a^ Step 1 of hierarchical regression of the association between each drug alert independent variable on behaviour in isolation.

^b^ Step 2 controlling for gender, age, gender*age, lifetime MDMA use, ever had an MDMA overdose, and seen public drug alert in the last 12 months. Age squared was included but was removed as *p* > 0.25.

^c^ Step 3 controlling for gender, age, gender*age, lifetime MDMA use, ever had an MDMA overdose, seen public drug alert in the last 12 months, and the other two categorical independent variables (these differ for each alert content condition, for example, for the analysis examining the effect of dose, the additional variables at Step 3 are the Description of Effects and the Description of actions variables, and so on).

## 4 DISCUSSION

The aim of the present study was to explore whether exposure to a drug alert about high‐dose MDMA, and variation in the content of that alert, was associated with MDMA use intentions among people who use MDMA. Analyses showed that, in a scenario of being in possession of MDMA pills/tablets that may be high‐dose, exposure to a drug alert was associated with self‐reported intention to not use the drug, versus using but reducing their initial dose, or using a similar or greater initial dose. Variation in the description of dose, effects and actions to reduce harm was not associated with intended consumption behaviour. These findings support the important role of drug alerts in communicating about higher‐risk substances and promoting engagement in harm reduction behaviours.

In both alert and control conditions in the present study, people were presented with a scenario of possessing a drug that ‘may be two times stronger than normal’. Analyses showed that most participants in both conditions reported they would not use the drug or would reduce their initial dose in such a scenario. This finding is supported by prior research with people who engage in drug checking, showing that people who receive chemical analysis results yielding a higher dose of MDMA than expected are more likely to report intending to reduce MDMA dose or not use MDMA [29]. Overall, this evidence highlights the impact that information around dose can have on future consumption behaviour and reinforces the importance of services such as drug checking in providing objective insight into drug composition in the absence of a regulated market.

The current findings also reinforce the potential impact of alerting people explicitly to potential risk through public notices. Those who received the alert were more likely to report an intention not to use the drug, rather than using but reducing their initial dose, or using the same or a greater typical dose. These results suggest that, while information about the dose is critical in encouraging harm reduction behaviour, issuing of an alert may have some additional impact in promoting more cautious behaviour when using a drug of concern. With early warning systems increasingly being established across Australian states and territories and internationally [32, 41, 42, 43, 44], the current study lends support to the issuing of public notices around circulating higher‐risk substances, particularly in areas where people may not yet have access to drug checking services to obtain information on contents and dose. This is not to say that drug alerts will be effective in encouraging harm reduction behaviour for all drug types (e.g., heroin, methamphetamine) or population groups. For example, some groups, particularly young men, may be prompted to engage in drug use that is constructed as more risky or dangerous, as a form of resistance to public health messages or scepticism of them [45, 46]. Similarly, previous work has shown that alerts related to ‘high potency heroin’ have sometimes led to increased drug‐seeking and overdose risk [37, 47]. Overall, these findings suggest the need for a multifaceted approach, including drug checking and communication of drug alerts, but also an appreciation that these strategies may have unintended consequences or little impact for some people. Study of the impact of alerts about different substances on different audiences, and the interplay of broader individual, social and structural factors, will be critical to informing public health communication moving forward.

Manipulation of drug alert information regarding descriptions of dose, effects and actions to reduce harm was not associated with a change in intentions around initial dosing. These findings suggest that the alert itself, rather than the nuance of these features, may be the most important aspect in potential behaviour. However, this requires further exploration, as it may be that the changes in messaging used in the alerts were not particularly salient to people, or that manipulation of other aspects of the alert—such as imagery of the drug, who communicates it (e.g., government versus peer‐led agency), and how it is communicated (e.g., social media versus face‐to‐face)—may lead to different findings. A similar argument applies to varying contexts of use, such as receipt of the alert in close proximity versus more distal to intended use, and the role of factors such as police presence and low drug availability [22, 48]. The present findings also should not be used to infer that the specifics of the drug alert content are not important. Indeed, previous research has shown the critical role of people who use drugs in the design and communication of drug alerts to ensure they are appropriate for, and salient to, the intended audience, and to minimise the risk of unintended consequences (e.g., increased perceived desirability of the drug of concern) [31, 33, 34, 35, 36].

### 4.1 Strengths and limitations

This study had several strengths. The sample was relatively large, participants were randomised to conditions, and the drug alert used was based on public notices recently released in Australia [49] to ensure the applicability of findings to practises at the time of the study. However, asking people who use MDMA to report on intended use behaviours may not be an accurate reflection of how they would actually behave when in possession of high‐dose MDMA. This is due to many aspects of drug behaviour influenced by ‘setting’ (i.e., social environment) that are not considered when asked to reflect upon how one might behave in a particular scenario [50]. Despite this, prior research examining MDMA use intentions and behaviour through the theory of planned behaviour showed that the intention to use MDMA predicted one's subsequent use behaviour over the following 2‐month [51] and 6‐month [52] periods.

It is important to note that this was a sample generally experienced in the use of MDMA; two‐thirds had used MDMA on more than 10 occasions. While lifetime MDMA use was controlled in the current analyses, it may be that this sample was more responsive to information about high‐dose MDMA and paid less attention to details about effects and actions to reduce harm—because they already are experienced in these aspects. As shown in Figure S1, there was a significant amount of drop‐off of survey respondents who began completing the survey, versus those who supplied all required data points to be included in the analytic sample. This potential sampling bias towards individuals willing to continue the survey until completion of core items, plus the use of a convenience sample, may also limit generalisability of findings [53]. Finally, self‐report data may be subject to bias; however, previous studies demonstrate sufficient reliability and validity of self‐reported drug use and related behaviour [54].

While not a limitation specific only to our study, a broader limitation of drug alerts based on pressed pills (as used in our example alert) is an underlying assumption that: (i) pills of similar appearance will be from the same batch that was tested; and (ii) that pills within the same batch have the same composition. These assumptions should be questioned, given that similarly looking batches may contain different ingredients or different doses [55, 56, 57].

## 5 CONCLUSION

This study shows that people exposed to a drug alert about high‐dose MDMA are more likely to report an intention not to use such a drug relative to those who were in a scenario of possessing high‐dose MDMA but who did not receive the alert. Manipulation of information on dose, effects and actions to reduce harm was not associated with a differential response. Overall, these findings suggest that drug alerts about higher‐risk substances may be an effective tool for government and other health agencies to encourage harm reduction behaviour among people who use drugs, particularly when employed as part of a multi‐faceted approach that includes drug checking and other harm reduction strategies. Testing manipulation of other aspects of the alert messaging and communication, responses to alerts around other drug types and actual behaviour following alert exposure, in collaboration with people who use drugs, are important next steps in understanding the potential outcomes of risk communication.

## AUTHOR CONTRIBUTIONS

All authors contributed to the conceptualisation of the data collection instrument and the study design. Joel Keygan and Breanna Willoughby undertook data collection. Joel Keygan, Breanna Willoughby and Monica J. Barratt undertook formal analyses. Joel Keygan prepared the first draft of the manuscript, and all authors reviewed, edited and approved the final version.

## CONFLICT OF INTEREST STATEMENT

Amy Peacock discloses an untied educational grant from Seqirus and Mundipharma for the study of opioid medications. Raimondo Bruno has received an untied educational grant from Mundipharma and Indivior for the study of opioid medications. Funding from these organisations has now ceased; the funding was for work unrelated to this project, and the funding bodies had no role in study design, analysis, and reporting. Monica J. Barratt is a volunteer for The Loop Australia, a charity that delivers drug checking services in Australia, including the issuing of public drug alerts. All other authors have no conflicts of interest to declare.

## Supporting information


**Data S1** Supporting information.

## Endnotes

^1^
At the time of data collection, there were no fixed site drug checking services in Australia. Subsequently, CanTEST—Canberra's fixed site drug checking service—was established in 2022, and Queensland's CheQpoint began operating in 2024 in Brisbane and the Gold Coast. Victoria's fixed site is due to begin operating in July 2025.

^2^
The Loop Australia was used as an example organisation for the purpose of the hypothetical alert. The Loop Australia is a not‐for‐profit organisation that, at the time, was working towards delivering drug checking in Australia, and has since done so in Queensland and Victoria. However, it should be noted that The Loop Australia was mistakenly described in this hypothetical alert as a peer‐based organisation representing people who use drugs.

## References

[1] De la Torre R , Farré M , Roset PN , Pizarro N , Abanades S , Segura M , et al. Human pharmacology of MDMA: pharmacokinetics, metabolism, and disposition. Ther Drug Monit. 2004;26:137–144. 15228154 10.1097/00007691-200404000-00009

[2] Sutherland R , Chandrasena U , Karlsson A , Uporova J , Tayeb H , Price O , et al. Australian drug trends 2024: key findings from the National Ecstasy and related drugs reporting system (EDRS) interviews. Sydney: National Drug and Alcohol Research Centre, UNSW Sydney; 2024.

[3] Brunt TM , Koeter MW , Niesink RJ , van den Brink W . Linking the pharmacological content of ecstasy tablets to the subjective experiences of drug users. Psychopharmacology. 2012;220:751–762. 21993879 10.1007/s00213-011-2529-4

[4] Giné CV , Vilamala MV , Espinosa IF , Lladanosa CG , Álvarez NC , Fruitós AF , et al. Crystals and tablets in the Spanish ecstasy market 2000–2014: are they the same or different in terms of purity and adulteration? Forensic Sci Int. 2016;263:164–168. 27129144 10.1016/j.forsciint.2016.04.016

[5] Sevigny EL , Thyssen S , Erowid E , Lea R . Misrepresentation of MDMA in the United States, 1999–2023. Drug Alcohol Depend. 2024;264:112467. 39437494 10.1016/j.drugalcdep.2024.112467

[6] Roxburgh A , Sam B , Kriikku P , Mounteney J , Castanera A , Dias M , et al. Trends in MDMA‐related mortality across four countries. Addiction. 2021;116:3094–3103. 33739562 10.1111/add.15493

[7] Hall AP , Henry JA . Acute toxic effects of ‘Ecstasy’ (MDMA) and related compounds: overview of pathophysiology and clinical management. Br J Anaesth. 2006;96:678–685. 16595612 10.1093/bja/ael078

[8] Rigg KK , Sharp A . Deaths related to MDMA (ecstasy/molly): prevalence, root causes, and harm reduction interventions. J Subst Use. 2018;23:345–352.

[9] Wolfgang AS , Fonzo GA , Gray JC , Krystal JH , Grzenda A , Widge AS , et al. MDMA and MDMA‐assisted therapy. Am J Psychiatry. 2025;182:79–103. 39741438 10.1176/appi.ajp.20230681

[10] Ritchie OD , Donley CN , Ritchie GD . From prohibited to prescribed: the rescheduling of MDMA and psilocybin in Australia. Drug Sci Policy Law. 2023;9:20503245231198472.

[11] Palamar JJ , Sönmez İ . A qualitative investigation exploring why dance festivals are risky environments for drug use and potential adverse outcomes. Harm Reduct J. 2022;19:12. 35120530 10.1186/s12954-022-00598-5 PMC8817488

[12] Liechti ME , Holze F . Dosing psychedelics and MDMA. In: Barrett FS , Preller KH , editors. Disruptive psychopharmacology. Cham: Springer International Publishing; 2022. p. 3–21. 10.1007/7854_2021_270 34734392

[13] Psychonaut Wiki . MDMA. 2025. Available from: https://psychonautwiki.org/wiki/MDMA

[14] European Monitoring Centre for Drugs and Drug Addiction . European drug report 2024: Trends and Developments. 2024.

[15] Mounteney J , Griffiths P , Bo A , Cunningham A , Matias J , Pirona A . Nine reasons why ecstasy is not quite what it used to be. Int J Drug Policy. 2018;51:36–41. 29156401 10.1016/j.drugpo.2017.09.016

[16] Vrolijk RQ , Measham F , Quesada A , Luf A , Schori D , Radley S , et al. Size matters: comparing the MDMA content and weight of ecstasy tablets submitted to European drug checking services in 2012–2021. Drugs Habits Soc Policy. 2022;23:207–219.

[17] O'Reilly MJ , Harvey CA , Auld R , Cretikos M , Francis C , Todd S , et al. A quantitative analysis of MDMA seized at New South Wales music festivals over the 2019/2020 season: form, purity, dose and adulterants. Drug Alcohol Rev. 2022;41:330–337. 34919770 10.1111/dar.13412

[18] Armenian P , Mamantov TM , Tsutaoka BT , Gerona RR , Silman EF , Wu AH , et al. Multiple MDMA (ecstasy) overdoses at a rave event: a case series. J Intensive Care Med. 2013;28:252–258. 22640978 10.1177/0885066612445982

[19] Roxburgh A , Lappin J . MDMA‐related deaths in Australia 2000 to 2018. Int J Drug Policy. 2019;76:102630. 31865118 10.1016/j.drugpo.2019.102630

[20] State Coroner's Court of New South Wales . Inquest into the death of six patrons of NSW music festivals. Lidcombe, NSW: NSW State Coroner's Court; 2019.

[21] Santamarina R , Caldicott D , Fitzgerald J , Schumann JL . Drug‐related deaths at Australian music festivals. Int J Drug Policy. 2024;123:104274. 38065009 10.1016/j.drugpo.2023.104274

[22] Fernández Calderón F , Díaz Batanero C , Barratt MJ , Palamar JJ . Harm reduction strategies related to dosing and their relation to harms among festival attendees who use multiple drugs. Drug Alcohol Rev. 2019;38:57–67. 30302851 10.1111/dar.12868 PMC6338512

[23] Bahora M , Sterk CE , Elifson KW . Understanding recreational ecstasy use in the United States: a qualitative inquiry. Int J Drug Policy. 2009;20:62–69. 18068967 10.1016/j.drugpo.2007.10.003 PMC2630386

[24] Span C , Farah B , Ivetìc N , Stronach O . A scoping review of Australian literature on people who use MDMA and their harm reduction practices. Contemp Drug Probl. 2024;51:25–44.

[25] Barratt MJ , Bruno R , Ezard N , Ritter A . Pill testing or drug checking in Australia: acceptability of service design features. Drug Alcohol Rev. 2018;37:226–236. 28635057 10.1111/dar.12576

[26] Akhurst J , Pierce A , Volpe I , Harrod ME , Sutherland R , Bruno R , et al. Informing drug alerts in Australia (IDAA) survey: awareness of, responses to, and preferences for communication of drug alerts. Drugs and new technologies (DNeT) bulletin Sydney: National Drug and Alcohol Research Centre, UNSW Sydney. 2024.

[27] Measham FC . Drug safety testing, disposals and dealing in an English field: exploring the operational and behavioural outcomes of the UK's first onsite ‘drug checking’ service. Int J Drug Policy. 2019;67:102–107. 30541674 10.1016/j.drugpo.2018.11.001

[28] Measham F . City checking: piloting the UK's first community‐based drug safety testing ('drug checking') service in two city centres. Br J Clin Pharmacol. 2020;86:420–428. 32030770 10.1111/bcp.14231 PMC7080630

[29] Measham F , Turnbull G . Intentions, actions and outcomes: a follow up survey on harm reduction practices after using an English festival drug checking service. Int J Drug Policy. 2021;95:103270. 33972157 10.1016/j.drugpo.2021.103270

[30] Peacock A , Gibbs D , Price O , Barratt MJ , Ezard N , Sutherland R , et al. Profile and correlates of colorimetric reagent kit use among people who use ecstasy/MDMA and other illegal stimulants in Australia. Int J Drug Policy. 2021;97:103334. 34246017 10.1016/j.drugpo.2021.103334

[31] Soukup‐Baljak Y , Greer AM , Amlani A , Sampson O , Buxton JA . Drug quality assessment practices and communication of drug alerts among people who use drugs. Int J Drug Policy. 2015;26:1251–1257. 26205676 10.1016/j.drugpo.2015.06.006

[32] Brien R , Volpe I , Grigg J , Lyons T , Hughes C , McKinnon G , et al. Co‐designing drug alerts for health and community workers for an emerging early warning system in Victoria, Australia. Harm Reduct J. 2023;20:30. 36894933 10.1186/s12954-023-00761-6 PMC9995746

[33] Freestone J , Franklin E , Broadbent E , Brown J , Harvey C , Barratt MJ , et al. Emerging best practices in the design and dissemination of public drug warnings. Sydney: National Centre for Clinical Research on Emerging Drugs, UNSW Sydney; 2025.

[34] British Columbia Centre for Disease Control (BCCDC) . Toxic Drug & Health Alerts: Guiding Principles. 2024.

[35] European Monitoring Centre for Drugs and Drug Addiction . Health risk communication strategies for drug checking services: a manual. Luxembourg: Publications Office of the European Union; 2023.

[36] Public Health England . Drug alerts and local drug information systems. London, UK: Public Health England; 2016. Available from: https://assets.publishing.service.gov.uk/media/5a7483b440f0b616bcb1717c/Drug_alerts_and_local_drug_information_systems_guidance.pdf

[37] Kerr T , Small W , Hyshka E , Maher L , Shannon K . ‘It's more about the heroin’: injection drug users' response to an overdose warning campaign in a Canadian setting. Addiction. 2013;108:1270–1276. 23551565 10.1111/add.12151 PMC3913056

[38] Waye KM , Yedinak JL , Koziol J , Marshall BDL . Action‐focused, plain language communication for overdose prevention: a qualitative analysis of Rhode Island's overdose surveillance and information dashboard. Int J Drug Policy. 2018;62:86–93. 30384027 10.1016/j.drugpo.2018.08.010

[39] The Know. The Australian emerging drug information and resource hub. 2025. Available from: https://theknow.org.au/

[40] Hosmer DW , Lemeshow S . Applied logistic regression. 2nd ed. New York, NY: Wiley; 2000.

[41] Camilleri A , Alfred S , Gerber C , Lymb S , Painter B , Rathjen A , et al. Delivering harm reduction to the community and frontline medical practitioners through the south Australian drug early warning system (SADEWS). Forensic Sci Med Pathol. 2021;17:388–394. 34013465 10.1007/s12024-021-00381-1

[42] Cottler LB , Goldberger BA , Nixon SJ , Striley CW , Barenholtz E , Fitzgerald ND , et al. Introducing NIDA's new National Drug Early Warning System. Drug Alcohol Depend. 2020;17:108286. 10.1016/j.drugalcdep.2020.108286 PMC7489265 32979739

[43] McCutcheon D , Raghavan M , Soderstrom J , Oosthuizen F , Douglas B , MacDonald E , et al. An early warning system for emerging drugs of concern in the emergency department: protocol for the Western Australian illicit substance evaluation (WISE) study. Emerg Med Australas. 2019;31:411–416. 30318770 10.1111/1742-6723.13185

[44] Syrjanen R , Schumann J , Fitzgerald J , Gerostamoulos D , Abouchedid R , Rotella J‐A , et al. The emerging drugs network of Australia – Victoria clinical registry: a state‐wide illicit substance surveillance and alert network. Emerg Med Australas. 2023;35:82–88. 36053993 10.1111/1742-6723.14059

[45] Barratt MJ , Allen M , Lenton S . ‘PMA sounds fun’: negotiating drug discourses online. Subst Use Misuse. 2014;49:987–998. 24779498 10.3109/10826084.2013.852584

[46] Farrugia A , Fraser S . Science and scepticism: drug information, young men and counterpublic health. Health (London). 2017;21:595–615. 26865213 10.1177/1363459315628042

[47] Dietze PM , Jolley D , Fry CL , Bammer G , Moore D . When is a little knowledge dangerous? Circumstances of recent heroin overdose and links to knowledge of overdose risk factors. Drug Alcohol Depend. 2006;84:223–230. 16542798 10.1016/j.drugalcdep.2006.02.005

[48] Grigg J , Barratt MJ , Lenton S . Double dropping down under: correlates of simultaneous consumption of two ecstasy pills in a sample of Australian outdoor music festival attendees. Drug Alcohol Rev. 2018;37:851–855. 30392182 10.1111/dar.12843

[49] NSW Government . Drug warning ‐ variable dose and high dose MDMA tablets/capsules. 2019. Available from: https://www.health.nsw.gov.au/aod/public‐drug‐alerts/Pages/2019.aspx

[50] Hartogsohn I . Constructing drug effects: a history of set and setting. Drug Sci Policy Law. 2017;3:2050324516683325.

[51] Orbell S , Blair C , Sherlock K , Conner M . The theory of planned behavior and ecstasy use: roles for habit and perceived control over taking versus obtaining substances. J Appl Soc Psychol. 2001;31:31–47.

[52] Valente H , Martins D , Pinto M , Fernandes L , Barratt MJ . A longitudinal study of behavioural outcomes following a visit to the boom festival 2018 drug checking service: individual and group level results. Drugs Educ Prev Policy. 2023;30:373–382.

[53] Bland JM , Altman DG . Multiple significance tests: the Bonferroni method. BMJ. 1995;310:170. 7833759 10.1136/bmj.310.6973.170 PMC2548561

[54] Bharat C , Webb P , Wilkinson Z , McKetin R , Grebely J , Farrell M , et al. Agreement between self‐reported illicit drug use and biological samples: a systematic review and meta‐analysis. Addiction. 2023;118:1624–1648. 37005867 10.1111/add.16200

[55] Duterte M , Jacinto C , Sales P , Murphy S . What's in a label? Ecstasy sellers' perceptions of pill brands. J Psychoactive Drugs. 2009;41:27–37. 19455907 10.1080/02791072.2009.10400672 PMC2717897

[56] Pascoe MJ , Radley S , Simmons HTD , Measham F . The cathinone hydra: increased cathinone and caffeine adulteration in the English MDMA market after Brexit and COVID‐19 lockdowns. Drug Sci Policy Law. 2022;8. 10.1177/20503245221099209

[57] Couchman L , Frinculescu A , Sobreira CC , Shine T , Ramsey J , Hecht M , et al. Variability in content and dissolution profiles of MDMA tablets collected in the UK between 2001 and 2018 – a potential risk to users? Drug Test Anal. 2019;11:1172–1182. 31009168 10.1002/dta.2605

