# Supplementary material for: Risk communication about high‐dose MDMA: Impact of a hypothetical drug alert on future MDMA use
Source: Drug Alcohol Rev. 2025 Mar 7;44(4):1169–81. doi: 10.1111/dar.14037 (PMC12117305; doi:10.1111/dar.14037)
Supplement: Supplementary file 1 — Data S1 Supporting information. [file DAR-44-1169-s001.docx]

**Appendix*:* Risk communication about high-dose MDMA: Impact of a hypothetical drug alert on future MDMA use**

**Figure S1. Sample inclusion process**

Participants who started the survey (n=2146)

Participants screened for eligibility (n=1941)

INELIGIBLE (n=277)

- Age < 18 years old (n=69)
- Lives outside Australia (n=22)
- No MDMA use in past 12 months (n=156)
- No pills/caps in the last 12 months (n=30)

Eligible sample (n=1664)

MISSING/DON’T KNOW (n=1039)

- Both outcome variables missing – indicates survey drop out prior to outcome measurement (n=685)
- Don’t know typical initial MDMA dose (n=10)
- Don’t know what they would do after seeing alert / high-dose scenario (n=80)
- Will use drug but missing dose information (n=3)
- Outcome variable is ‘no, prefer crystal/powder’ (n=241)
- Missing lifetime MDMA experience (n=10)
- Non-binary or prefer not to say gender (n=10) *

Final sample (n=625)

* n=10 with non-binary or missing gender was too small a group to include in regression analyses.

**Table S1. Covariates**

| Covariates | Measurement | Rationale for inclusion |
| --- | --- | --- |
| Gender | 1 = male, 2 = female (gender fluid/non-binary and prefer not to say = captured but excluded from analysis due to small numbers). | Males are more likely than females to use harm reduction strategies (including dose reduction), take larger doses, and have greater risk-taking attitudes towards MDMA [1-3]. Furthermore, Kaye et al. [4] observed that the majority of MDMA-related deaths as a direct of antecedent cause occurred among males (83%). |
| Age | Continuous | Being younger is a risk factor for more ecstasy related problems (negative effects) and risky behaviours such as ‘double dropping’ [3, 5]. This indicates that younger people may be less likely to use and reduce initial dose or not use high-dose MDMA. |
| Lifetime frequency of MDMA use | 1-10 times (reference group), 11-50 times, 51+ times | People with greater lifetime use of ecstasy (50+ times) are more likely to alter use to reduce negative effects [1]. Alternatively, Fernández-Calderón et al. [2] observed than people who had more recently commenced use were more likely to adopt harm reduction strategies, while Grigg et al. [3] found those who used more frequently were more likely to ‘double drop’. |
| Experienced MDMA non-fatal overdose in lifetime | Yes, no. Overdose was defined as “An MDMA overdose is where you experience negative effects (ranging from jaw clenching and excessive sweating to paranoia and seizures) and/or require professional help.” | Experiencing negative an MDMA overdose from MDMA may alter their use to reduce these negative effects [1]. This was controlled for to determine whether participants who have experienced an overdose are more likely to reduce their initial dose of MDMA suspected to be high dose compared to participants who say they have not experienced an overdose. |
| Previously seen any public notices (drug alerts) about high-dose MDMA/ecstasy in the past 12 months | Yes, no | There is a theoretical basis to include this as a covariate. Soukup-Baljak et al. (6) state that drug alerts can desensitise people who use drugs to warnings provided, meaning that they dismiss the dangerousness of high-dose MDMA, affecting their health beliefs, and how they would respond if in possession of high-dose MDMA. |

**Table S2: Descriptive statistics across outcomes groups for each covariate measure (%; n=625)**

| Co-variate | No use | Use and reduce initial dose | Use and do not reduce initial dose | Χ2, *p*^a^ |
| --- | --- | --- | --- | --- |
| Gender |  |  |  |  |
| Male | 44.5 | 66.1 | 74.1 | χ2 = 32.37, *p* <0.001 |
| Female | 55.5 | 33.9 | 25.9 |  |
| Age |  |  |  |  |
| Median (IQR) | 22 (20-25) | 22 (19-25) | 21 (19-23) | χ2 = 0.96, *p*=0.618 |
| Age group x gender |  |  |  |  |
| 18-20yo males | 16.0 | 27.9 | 29.6 | χ2 = 43.91, *p* <0.001 |
| 21-23yo males | 13.9 | 19.8 | 27.8 |  |
| 24+yo males | 14.7 | 18.3 | 16.7 |  |
| 18-20yo females | 18.9 | 13.2 | 14.8 |  |
| 21-23yo females | 18.1 | 6.3 | 7.4 |  |
| 24+ females | 18.5 | 14.4 | 3.7 |  |
| Lifetime MDMA use |  |  |  |  |
| 1-10 times | 46.6 | 24.0 | 22.2 | χ2 = 45.64, *p* <0.001 |
| 11-50 times | 37.4 | 46.9 | 33.3 |  |
| 51+ times | 16.0 | 29.1 | 44.4 |  |
| Ever had MDMA overdose |  |  |  |  |
| No/don’t know | 68.9 | 64.6 | 63.0 | χ2 = 1.42, *p*=0.491 |
| Yes | 31.1 | 35.4 | 37.0 |  |
| Seen public drug alert in the last 12 months |  |  |  |  |
| No/don’t know | 68.1 | 67.9 | 75.9 | χ2 = 1.46, *p*=0.482 |
| Yes | 31.9 | 32.1 | 24.1 |  |

*Note.* ^a^All comparisons except age were categorical. Chi square tests of association were conducted. For age, a median test was conducted. For the interaction of age group and gender, this has been simplified into 6 categories here for ease of reporting descriptively, however in all subsequent regression analyses, the age variable was treated as continuous (age and age*gender).

IQR, interquartile range.

**Table S3: Hierarchical binary logistic regression of association between alert content and Intended MDMA Use/Initial Dose (n=406)**

|  |  | Changed dose (DV) | | Hierarchical logistic regression steps | | |
| --- | --- | --- | --- | --- | --- | --- |
| Variables | Total sample  (n=406)  % (n) | No use (ref)  49.3% (n=200)  % (n) | Use and reduce initial dose  50.7% (n=206)  % (n) | Step 1^a^  OR (95% CI, *p*) | Step 2^b^  AOR (95% CI, *p*) | Step 3^c^  AOR (95% CI, *p*) |
| **Description of dose** |  |  |  |  |  |  |
| “High dose MDMA” | 31.8 (129) | 31.5 (63) | 32.0 (66) | – | – | – |
| “High dose MDMA 200mg” | 32.8 (133) | 31.5 (63) | 34.0 (70) | 1.06 (0.65-1.72, 0.812) | 1.24 (0.74-2.01, 0.414) | 1.24 (0.73-2.09, 0.425) |
| “High dose MDMA 200mg 2-3x standard dose” | 35.5 (144) | 37.0 (74) | 34.0 (70) | 0.90 (0.56-1.45, 0.674) | 1.11 (0.66-1.86, 0.688) | 1.11 (0.66-1.86, 0.703) |
| **Description of effects** |  |  |  |  |  |  |
| Mild | 49.3 (200) | 50.5 (101) | 48.1 (99) | – | – | – |
| Severe | 50.7 (206) | 49.5 (99) | 51.9 (107) | 1.10 (0.75-1.63, 0.623) | 1.07 (0.70-1.63, 0.766) | 1.06 (0.70-1.62, 0.778) |
| **Description of actions** |  |  |  |  |  |  |
| Emergency | 35.5 (144) | 35.0 (70) | 35.9 (74) | – | – | – |
| Emergency + do not use | 32.0 (130) | 32.5 (65) | 31.6 (65) | 0.95 (0.59-1.52, 0.818) | 0.94 (0.57-1.57, 0.817) | 0.94 (0.57-1.57, 0.818) |
| Emergency + harm reduction | 32.5 (132) | 32.5 (65) | 32.5 (67) | 0.98 (0.61-1.56, 0.917) | 1.03 (0.62-1.72, 0.904) | 1.03 (0.62-1.71, 0.919) |
| Gender |  |  |  |  |  |  |
| Female | 41.3 (97) | 59.2 (61) | 27.3 (36) | – | – | – |
| Male | 58.7 (138) | 40.8 (42) | 72.7(96) | – | **30.96 (4.13-232.05, 0.001)** | **31.81 (4.10-246.76, 0.001)** |
| Age ^a^ | – | – | – | – | 1.01 (0.86-1.16, 0.990) | 1.00 (0.87-1.17, 0.961) |
| Gender*age | – | – | – | – | **0.90 (0.83-0.98, 0.020)** | **0.90 (0.83-0.99, 0.022)** |
| Lifetime MDMA use |  |  |  |  |  |  |
| 1-10 times | 32.8 (77) | 43.7 (45) | 24.2 (32) | – | **2.11 (1.30-3.45, 0.003)** | **2.11 (1.30-3.45, 0.003)** |
| 11-50 times | 43.0 (101) | 38.8 (40) | 46.2 (61) | – | - | - |
| 51 times or more | 24.3 (57) | 17.5 (18) | 29.5 (39) | – | **3.16 (1.71-5.83, <0.001)** | **3.17 (1.71-5.86, <0.001)** |
| Ever had an MDMA overdose |  |  |  |  |  |  |
| No/don’t know | 65.5 (266) | 69.0 (138) | 62.1 (128) | **-** | – | – |
| Yes | 34.5 (140) | 31.0 (62) | 37.9 (78) | – | 1.27 (0.82-1.99, 0.289) | 1.28 (0.82-2.00, 0.283) |
| Seen public drug alert in last 12 months |  |  |  |  |  |  |
| No/don’t know | 68.0 (276) | 68.0 (136) | 68.0 (140) | – | – | – |
| Yes | 32.0 (130) | 32.0 (64) | 32.0 (66) | – | 0.87 (0.55-1.37, 0.545) | 0.86 (0.54-1.37, 0.523) |

*Note*. ^a^ Step 1 of hierarchical regression of the association between each drug alert independent variable on behaviour in isolation; ^b^ controlling for gender, age, gender*age, lifetime MDMA use, ever had an MDMA overdose, and seen public drug alert in last 12 months. Age squared was included but was removed as *p* >0.25.; ^c^ controlling for gender, age, gender*age, lifetime MDMA use, ever had an MDMA overdose, seen public drug alert in last 12 months, and other two independent variables. DV, dependent variable; OR, odds ratio; AOR, adjusted odds ratio (controlling for gender, gender*age, lifetime MDMA use, ever had an MDMA overdose, and seen public drug alert in last 12 months); CI, confidence interval. *p* <0.05 are bolded.

**References**

1. Allott K, Redman J. Patterns of use and harm reduction practices of ecstasy users in Australia. Drug Alcohol Depend. 2006;82:168-76.

2. Fernández-Calderón F, Díaz-Batanero C, Barratt MJ, Palamar JJ. Harm reduction strategies related to dosing and their relation to harms among festival attendees who use multiple drugs. Drug Alcohol Rev. 2019;38:57-67.

3. Grigg J, Barratt MJ, Lenton S. Double dropping down under: Correlates of simultaneous consumption of two ecstasy pills in a sample of Australian outdoor music festival attendees. Drug Alcohol Rev. 2018;37:851-5.

4. Kaye S, Darke S, Duflou J. Methylenedioxymethamphetamine (MDMA)-related fatalities in Australia: demographics, circumstances, toxicology and major organ pathology. Drug Alcohol Depend. 2009;104:254-61.

5. Baggott MJ. Preventing problems in ecstasy users: Reduce use to reduce harm. J Psychoactive Drugs. 2002;34:145-62.

6. Soukup-Baljak Y, Greer AM, Amlani A, Sampson O, Buxton JA. Drug quality assessment practices and communication of drug alerts among people who use drugs. Int J Drug Policy. 2015;26:1251-7.
